# Agent-Based Modeling of Chronic Diseases: A Narrative Review and Future Research Directions

**DOI:** 10.5888/pcd13.150561

**Published:** 2016-05-26

**Authors:** Yan Li, Mark A. Lawley, David S. Siscovick, Donglan Zhang, José A. Pagán

**Affiliations:** Author Affiliations: Mark A. Lawley, Center for Remote Health Technologies and Systems and Department of Industrial and Systems Engineering, Texas A&M University, College Station, Texas; David S. Siscovick, The New York Academy of Medicine, New York, New York; Donglan Zhang, Department of Health Policy and Management, University of California, Los Angeles, California; José A. Pagán, Center for Health Innovation, The New York Academy of Medicine, New York, New York, Department of Population Health Science and Policy, Icahn School of Medicine at Mount Sinai, New York, New York, Leonard Davis Institute of Health Economics, University of Pennsylvania, Philadelphia, Pennsylvania.

## Abstract

The United States is experiencing an epidemic of chronic disease. As the US population ages, health care providers and policy makers urgently need decision models that provide systematic, credible prediction regarding the prevention and treatment of chronic diseases to improve population health management and medical decision-making. Agent-based modeling is a promising systems science approach that can model complex interactions and processes related to chronic health conditions, such as adaptive behaviors, feedback loops, and contextual effects. This article introduces agent-based modeling by providing a narrative review of agent-based models of chronic disease and identifying the characteristics of various chronic health conditions that must be taken into account to build effective clinical- and policy-relevant models. We also identify barriers to adopting agent-based models to study chronic diseases. Finally, we discuss future research directions of agent-based modeling applied to problems related to specific chronic health conditions.

## Introduction

Americans are facing a growing burden of chronic disease that includes heart disease, stroke, diabetes, and obesity. By 2013, nearly half of the adult population in the United States had at least one chronic health condition, and approximately 70% of deaths were caused by chronic disease (1). The high prevalence of chronic disease is also a substantial economic burden to the nation. A 2007 report from the Milken Institute estimated that the total impact of chronic disease on the US economy was $1.3 trillion annually (2). As the retirement-aged population in the United States continues its rapid growth, health care providers and policy makers need to make informed decisions to effectively control the chronic disease epidemic and, thereby, improve population health and reduce health care costs.

Systems science methodologies such as system dynamics, discrete-event simulation, network analysis, and agent-based modeling have the potential to inform decision makers on how chronic health conditions develop and their consequences. Unlike standard statistical models, which often assume independence of observations, unidirectional causality, and noninterference, systems science methodologies allow for integration of data and evidence from many different sources and at many levels of analysis (3). However, compared with mainstream statistical methodologies and experimental studies, systems science methodologies are underused during the development of health policy, population health management, and clinical decision-making (3,4).

This article 1) introduces the concept of agent-based modeling — a relatively new systems science methodology, 2) reviews existing agent-based modeling of several prevalent chronic diseases (ie, diabetes, cardiovascular disease, obesity), 3) identifies barriers to adopting agent-based modeling to study chronic diseases, and 4) proposes novel research directions in this promising field that can lead to informed policy and practice.

## Agent-Based Modeling

Agent-based modeling is a computational modeling approach in which system-level emergent phenomena can be observed through explicit modeling of individual behaviors and their interactions with each other and with the environment (5,6). Agents (eg, individuals, organizations) in an agent-based model may be endowed with a large set of “real-world” properties (Table). Agent-based models can be used to uncover complex causal effects, identify underlying mechanisms behind complex systems, and make sense of large amounts of existing evidence and data. In addition, the fast growth of agent-based modeling software, such as NetLogo (The Center for Connected Learning and Computer-Based Modeling, Northwestern University), Repast (Argonne National Laboratory), Swarm (Swarm Development Group), and AnyLogic (The AnyLogic Company), has simplified the model development process and facilitated the use of agent-based modeling in various fields.

**Table:** Key Properties of Agent-Based Modeling of Chronic Diseases

Property	Description
Interactive	Agents can interact with each other or with the environment
Heterogeneous	Agents can have different attributes, states, or behaviors
Dynamic	Agents can change their attributes, states, or behaviors with time or location
Stochastic	Agents can decide their attributes, states, or behaviors based on probability distribution
Rational	Agents can act in their best interest based on their own knowledge and preference
Adaptive	Agents can change their states or behaviors based on the current state of the system
Autonomous	Agents can decide their own states or behaviors
Mobile	Agents can move in a geographic space
Memory	Agents can remember their previous attributes, states, and behaviors or the history of the system

In medicine and public health, state-transition models (eg, Markov models) have been widely used to capture disease progression and inform medical decision-making and public health intervention design (7). Markov models assume that the probability distribution of future states depends only on the present state and, thus, cannot reflect the fact that risk factors developed early in life affect future disease progression. Moreover, these models have significant limitations when risk factors and outcomes of the disease being studied exhibit complex properties such as adaptive behaviors (ie, people can change behaviors on the basis of the current state of the system), feedback loops (ie, causal effects can be reinforced or offset over time), and contextual effects (ie, individual health factors and outcomes are affected by social, cultural, and economic factors) (8,9). Agent-based modeling can integrate these complex properties and help elucidate interdependent causal effects and the impact of these interdependencies on population health (9–11).

Agent-based modeling also represents a promising approach to conducting counterfactual studies (9). In most observational studies, the analytical focus centers on a single intervention or exposure. A robust analysis of causal effects focuses on knowing what would have happened if a given intervention had not been implemented or if a different intervention had been implemented. In agent-based modeling, agents can represent people who 1) have heterogeneous demographic characteristics (eg, age, sex, race/ethnicity) and behaviors (eg, smoking, having an unhealthful diet), 2) exhibit endogenous evolution of health conditions (eg, having elevated blood pressure, developing type 2 diabetes) and exogenous interactions (eg, transmission of infectious disease, diffusion of health information), and 3) live in a certain geographic location or participate in a virtual social environment (eg, Facebook). By using agent-based modeling, investigators can predict counterfactual outcomes of any intervention or no intervention on the same population in the same physical and social environment. The simulated results enable researchers to identify causal effects between risk factors and health outcomes and compare the effectiveness of interventions.

Agent-based modeling applications are much more common in the study of infectious diseases (eg, influenza, sexually transmitted diseases) than chronic diseases (12). One reason is that infectious diseases have a clear path of disease transmission characterized by nonlinear, stochastic, and dynamic interactions between human beings and the environment. These interactive and complex features cannot be captured with traditional statistical models or models based on differential equations (13). By generating populations of different sizes and incorporating geographic location information, agent-based modeling provides the flexibility to model disease transmissions at different scales from local to global and to examine the impact of alternative interventions. For example, Lee et al developed an agent-based model of the H1N1 influenza to design vaccination allocation strategies (14).

In addition to modeling infectious diseases, agent-based modeling has been used to assess different strategies designed to combat addictive behaviors such as alcohol use and smoking (15,16). It is worth noting that the US Food and Drug Administration, working with the Institute of Medicine, has explored agent-based modeling as a way to inform its policy decisions on tobacco control (11). Because smoking is a major risk factor for many chronic diseases, developing agent-based models of chronic disease may be a natural next step. However, such models are still rare.

Nianogo et al recently conducted a systematic review of agent-based modeling of noncommunicable diseases and underscored the importance of using agent-based modeling to inform design of public health interventions (17). However, 14 out of the 22 studies those authors reviewed modeled only health behaviors (eg, walking, alcohol use, diet, smoking) and not chronic disease progression. With a focus on specific chronic diseases (ie, diabetes, cardiovascular disease, obesity), we searched PubMed (Medline) by using a combination of keywords from 2 categories: 1) agent-based model or individual-based model, and 2) heart disease, cardiovascular disease, diabetes, or obesity. We did not conduct a systematic review, because the body of evidence was too sparse after we excluded models of behaviors. Another difference between this study and that of Nianogo et al is that we compare agent-based modeling with other modeling approaches in general and point out future research directions with regard to specific chronic diseases (17).

## Examples of Agent-Based Models of Chronic Diseases

### Diabetes

Diabetes is a metabolic disease caused by the interplay among many exogenous and endogenous factors (eg, lifestyle, genes, environment) that leads to complications and adverse health outcomes (eg, retinopathy, neuropathy, nephropathy, myocardial infarction, stroke, death). The prevalence of diabetes in the United States is projected to increase from 9.3% in 2012 to 33% by 2050 (18). In addition, diabetes costs the country approximately $245 billion per year (19). Both Markov-based models and system dynamics models have been developed to study the progression of diabetes and its complications and the impact of interventions (20,21).

Day et al provided an example of studying diabetes with agent-based modeling (22). In particular, they developed an agent-based modeling template for diabetic retinopathy, a common diabetes-related complication and the leading cause of blindness among US adults. Agents in their model are heterogeneous patients with a range of attributes — age, sex, smoking status, body mass index (BMI), HemoglobinA1c (glycated hemoglobin) level, duration of diabetes, hypertension, high cholesterol, diabetic nephropathy, and current status of nonproliferative diabetic retinopathy and proliferative diabetic retinopathy. They used longitudinal patient data (from 2006–2010) extracted from the eye clinic of the Veterans Administration St. Louis Healthcare System to calibrate model parameters and conduct predictive validation. They used agent-based modeling to assess the impact of different screening intervals on the incidence of vision loss among a simulated cohort of veterans and found that a screening interval of 2 years was the most reasonable and should be adopted (23).

### Cardiovascular disease

Cardiovascular disease is the leading cause of death in the United States (24). The total direct medical cost of cardiovascular disease is projected to increase from $273 billion in 2010 to $818 billion in 2030 (24). Unal et al conducted a systematic literature review of coronary heart disease policy models and found all the models were Markov models (25). Hirsch et al developed a system dynamics model for cardiovascular disease and used the model to evaluate the effectiveness of various interventions (26). However, their model was unable to capture the impact of heterogeneous populations on the effectiveness of different interventions, limiting the generalizability of the findings to other populations.

To overcome the limitations associated with Markov models and system dynamics models when assessing cardiovascular disease progression, Li et al developed an agent-based model of cardiovascular disease and used the model to evaluate the impact of several lifestyle interventions —quitting smoking, increasing physical activity, promoting healthy diet, and reducing weight — on the long-term prevalence and incidence of myocardial infarction and stroke for populations across different age groups or geographic locations (27,28). In their model, each agent (person) was defined according to 7 key behaviors or health factors (ie, smoking, physical activity, diet, weight, cholesterol, blood pressure, and blood glucose) and by age, sex, and whether the person had a history of myocardial infarction or stroke. These factors were selected on the basis of the concept of ideal cardiovascular health developed by the American Heart Association (29). Each agent’s behaviors and health factors evolve simultaneously and interactively as time progresses in the model. The model was validated by using data from the 2007 Behavioral Risk Factor Surveillance System (http://www.cdc.gov/brfss/annual_data/annual_2007.htm) and the 2012 Behavioral Risk Factor Surveillance System (http://www.cdc.gov/brfss/annual_data/annual_2012.html). The authors showed that a prevention intervention may have different effects on populations in different geographical areas; for example, a hypothetical lifestyle intervention promoting healthful diet, physical activity, and smoking cessation may reduce the proportion of the population with diabetes more significantly in the Buffalo-Cheektowaga-Tonawanda Metropolitan Statistical Area than it would in New York City (27). Thus, local health departments need to take into account their population characteristics and health profiles when prioritizing prevention interventions.

### Obesity

Obesity, defined as a body mass index (BMI) (kg/m^2^) of 30 or greater, is a chronic condition and also an important risk factor for many other chronic diseases, including hypertension, hypercholesterolemia, type 2 diabetes, asthma, myocardial infarction, and stroke. The prevalence of obesity among US adults increased significantly from 2000 through 2010 and reached about 36.5% from 2011 through 2014 (30). The annual direct medical costs associated with overweight and obesity in 2008 was estimated to be nearly $114 billion, which accounted for approximately 5% to 10% of US health care spending (31). The change in a person’s BMI is a complex process characterized by interactions among multiple biologic, behavioral, socio-economic, environmental, and cultural factors. Levy et al provided a detailed review of 14 simulation models of obesity (32).

Hammond and Ornstein developed an agent-based model to explicitly capture the impact of social influence on body weight (33). In their model, social influence changes each individual’s BMI on the basis of the theory of “follow the average” (34). The authors validated their simulation results by using data from a longitudinal survey of American youth (ie, National Longitudinal Survey of Youth 1997 cohort [https://www.nlsinfo.org/content/cohorts/nlsy97]). Similarly, Zhang et al developed an agent-based model to examine the impact of social influence on adolescent overweight and obesity (35). They compared 5 adolescent social network-related interventions and found that strengthening peer influence may be effective to combat obesity in populations with low obesity prevalence. Finally, El-Sayed et al built an agent-based social network model of obesity for the population of England to study the effectiveness of interventions targeting highly networked individuals (36). By using data from the Health Surveys for England in 1999 and 2004 (https://www.ucl.ac.uk/hssrg/studies/hse), they found that interventions targeting highly networked individuals were no more likely to reduce obesity prevalence than were interventions targeting random populations.

## Future Research Directions

Research using agent-based modeling to study chronic diseases is still in its infancy. We provided 3 possible reasons for a low adoption rate of agent-based modeling in the study of chronic health conditions and their consequences. First, chronic diseases are not characterized by clear “transmission” mechanisms; thus, many researchers are reluctant to use agent-based modeling to study chronic diseases because of the general perception that agent-based modeling is only suitable to model health conditions that can be transmitted from person to person. Second, it is generally more difficult to implement agent-based modeling than more widely used simulation approaches such as Markov-based state-transition models. In most cases, developing an agent-based model requires some training in computer programming, whereas constructing Markov-based models can be done using spreadsheet software (eg, Microsoft Excel) or specialized, easy-to-use software such as TreeAge Pro (TreeAge Software, Inc). Finally, the development of agent-based models generally requires a large amount of individual-level data for parameterization, calibration, and validation; such data are not always available to researchers. Despite these barriers, we believe that policymakers and health care providers would benefit from having access to high-quality, well-designed agent-based models that can help them better understand the development and consequences of chronic diseases and thereby improve their decision-making with regard to intervention design and resource allocation.

### Disease-specific future research directions

**Diabetes.** To the best of our knowledge, agent-based modeling has only been applied to the study of diabetic retinopathy (22,23). However, we believe that it can also be useful to study the progression of other diabetic complications — nephropathy, neuropathy, myocardial infarction, and stroke. In addition, future agent-based models should incorporate health behaviors, such as diet, physical activity, and smoking, and examine the impact of modifying behaviors on the prevention and management of diabetes. Finally, agent-based modeling should take into account the impact of comorbidities (eg, obesity, hypertension, hyperlipidemia) and pharmacologic interventions on the health outcomes of a person with diabetes.

**Cardiovascular disease.** Although the model in the study by Li et al demonstrated the possibility of using an agent-based model to study cardiovascular disease, the model has some limitations related to its design and structure (27). For example, a person is either of normal weight or overweight in the model, and detailed changes in BMI are not modeled. We believe that an agent-based model of cardiovascular disease with more detailed disease progression and validated model prediction will provide potential users with more precise insights and more confidence in using the results to inform decision-making. In addition, we suggest incorporating social influence in future modeling when studying the impact of lifestyle interventions on cardiovascular disease. Finally, future agent-based models of cardiovascular disease could take into account the effects of different treatment strategies, drug therapies, and procedures (eg, revascularization, pacemaker implantation) to improve their clinical relevance.

**Obesity.** Most agent-based models of obesity focused on the impact of social influences (peer effects) on the dynamics of obesity (33,35). However, social influences may not be the only factors or the most important factors associated with obesity. We suggest incorporating health behaviors, such as physical activity and diet, in future agent-based models of obesity. Moreover, agent-based models of obesity could be more useful if they took into account evidence from biology, behavioral science, and psychology to better understand the development and progression of obesity.

**Multimorbidity.** Although multimorbidity has become the most common chronic condition among the elderly population (age 65 or older) in the United States (37), credible agent-based models studying the development and consequences of multimorbidity are lacking. Thus, modelers and interested public health and medical researchers should strive to develop comprehensive agent-based models of multimorbidity in which both the characteristics of individual chronic conditions as well as the possible interactions across these health conditions are explicitly captured.

### Purpose-specific future research directions

**Risk assessment.** Risk assessment for chronic disease is an essential component of population health management. Current risk assessment tools rely on standard statistical models (eg, regression) to identify correlations in somewhat limited administrative data sets. Even more advanced statistical methods, such as structural equation modeling and latent class analysis, are unable to capture the common nonlinearity, interdependency, and dynamics of risk factors and disease outcomes among the individuals that make up a population. Thus, a promising future research direction is to use agent-based models to assess the risk of chronic disease and disease-specific mortality. Agent-based models capture the development of chronic disease as an emergent outcome of a set of factors, including health beliefs, social norms, lifestyle behaviors, medication compliance, and biomarkers, that often change stochastically, dynamically, and interactively. As demonstrated in Li et al, an agent-based model of cardiovascular disease can be used to assess the risk of a population of interest and, potentially, can become an essential part of population health management (28).

**Cost-effectiveness analysis.** Most model-based cost-effectiveness analyses are based on Markov models. However, Markov models have been criticized for having many limitations and inherent assumptions that may render the results misleading (38). Examples of limitations for Markov models are its inability to model heterogeneous populations (ie, with a set of population characteristics) or to account for dependence on prior states of the system. A few studies have demonstrated that agent-based modeling can overcome some limitations of Markov models and provide decision-makers with more flexibility in studying the cost-effectiveness of a certain intervention to prevent chronic diseases (39,40). However, researchers have not fully taken advantage of the modeling power of agent-based models — such as capturing population interactions and integrating individual-level data — to improve the accuracy and credibility of cost-effectiveness analysis.

Although agent-based modeling is a powerful approach to studying chronic health conditions, it remains an underused tool among researchers in medicine and public health who are interested in chronic disease prevention and management. We provide examples of agent-based modeling applications in the areas of diabetes, cardiovascular disease, and obesity. The broader use of agent-based modeling has the potential to provide new insights in the areas of population health management, medical decision-making, and health care policy formulation and assessment.
